# Design of Small-Sized Spiral Slot PIFA Antenna Used Conformally in Laminated Body Tissues

**DOI:** 10.3390/s25092938

**Published:** 2025-05-07

**Authors:** Rong Li, Jian Liu, Cuizhen Sun, Wang Yao, Ying Tian, Xiaojun Huang

**Affiliations:** College of Communication and Information Engineering, Xi’an University of Science and Technology, Xi’an 710061, China; xli3000@xust.edu.cn (R.L.); liujian02@xust.edu.cn (J.L.); scz@xust.edu.cn (C.S.); yaowang@xust.edu.cn (W.Y.); tianying@xust.edu.cn (Y.T.)

**Keywords:** Laminated body tissue, PIFA, Slot antenna, Spiral antenna, Conformal antenna

## Abstract

This paper presents a novel Spiral Slot Planar Inverted-F Antenna (SSPIFA) specifically designed for telemedicine and healthcare applications, featuring compact size, biocompatible safety, and high integration suitability. By replacing the conventional top metal patch of a Planar Inverted-F Antenna (PIFA) with a slot spiral radiator whose geometry is precisely matched to the ground plane, the proposed antenna achieves a significant size reduction, making it ideal for encapsulation in miniaturized medical devices—a critical requirement for implantation scenarios. Tailored for the ISM 915 MHz band, the antenna is fabricated with a four-turn slot spiral etched on a 30 mm-diameter dielectric substrate, achieving an overall height of 22 mm and an electrically small profile of approximately 0.09λ × 0.06λ (λ: free-space wavelength at the center frequency). Simulation and measurement results demonstrate a −16 dB impedance matching (S11 parameter) at the target frequency, accompanied by a narrow fractional bandwidth of 1% and stable right-hand circular polarization (RHCP). When implanted in a layered biological tissue model (skin, fat, muscle), the antenna exhibits a near-omni directional radiation pattern in the azimuthal plane, with a peak gain of 2.94 dBi and consistent performance across the target band. These characteristics highlight the SSPIFA’s potential for reliable wireless communication in implantable medical systems, balancing miniaturization, radiation efficiency, and biocompatible design.

## 1. Introduction

The design of low-profile, compact, and electrically small antennas (ESAs) remains a long-standing research challenge, driven by both technological limitations and emerging application demands. Numerous prior studies have focused on reducing antenna size, with one of the most effective approaches being the transformation of conventional dipole topologies into inverted-F structures, which have been proven to reduce electrical length by approximately a quarter-wavelength [1,2]. Further evolution of this architecture replaces the linear top arm with a planar radiating patch, giving rise to the Planar Inverted-F Antenna (PIFA). This planar geometry offers increased design flexibility by enabling the integration of diverse parasitic elements within its structure, such as the embedded multi-layer dielectric substrates to modify resonant characteristics [3]; the stacked layered superstrates on the top surface to enhance radiation efficiency [4]; the additional resonant branches in adjacent spaces for multi-band operation [5]; and the etched apertures/slots on the radiating patch or ground plane to tailor impedance and polarization [6,7]. These modifications leverage the PIFA’s inherent compactness to balance miniaturization with performance, making it a versatile platform for modern wireless systems [8,9].

It has been found that etching U-slots on the top patch of a PIFA represents the most feasible way to achieve antenna miniaturization [10,11]. Slot-loading techniques, a cornerstone of modern antenna miniaturization, enable the original antenna topology to generate additional low-frequency resonances by introducing tailored slot geometries—such as circular slots, square slots, metasurface-loaded slots, and spiral slots. These configurations effectively reduce the antenna’s electrical size by altering current distribution, collectively enabling substantial size reductions without compromising radiation performance [12,13].

The drive to develop smaller, lower-profile antenna topologies arises from the need to meet rigorous demands of safe implantable applications, which impose strict constraints on antenna size, geometric configuration, material biocompatibility, and operational reliability [14,15,16]. A primary application scenario is telemedicine and remote healthcare systems, where implantable antennas are typically embedded within biological tissues to facilitate wireless transmission/reception of physiological signals and data for medical diagnostics or treatment [17,18]. These antennas play a critical role in ensuring safe, stable data transmission through layered human tissues—a challenging environment characterized by complex dielectric properties and limited spatial availability [19,20].

For implantable antennas, penetration capability through complex multi-dielectric media—such as the typically layered structure of human body tissues (skin, fat, and muscle)—is critical. The UHF band is widely recognized as optimal for telemedicine and remote healthcare applications due to its balance between tissue penetration depth and manageable bandwidth [21]. Additionally, transmission efficiency remains a key challenge, primarily influenced by ohmic losses incurred in metallic components and feeding structures [22,23].

Conventional Planar Inverted-F Antennas (PIFAs), even when reconfigured with spiral-shaped top patches, cannot simultaneously satisfy all design criteria for implantable devices: biocompatible safety, ultra-compact size, low profile, tissue embedability, omnidirectional radiation, minimal ohmic loss, and high efficiency. These competing requirements necessitate innovative structural modifications or material selections to bridge the gap between miniaturization and performance.

This paper presents the design and development of a Spiral Slot Planar Inverted-F Antenna (SSPIFA), where the key innovation involves replacing the conventional spiral patch with a slot-loaded structure. This modification aims to reduce metallic content, thereby minimizing ohmic losses in the radiating structure, and enhance structural adaptability, enabling better conformity to the constrained geometry of implantable applications.

The proposed SSPIFA is an electrically small antenna (ESA) with a compact profile of 0.09λ × 0.06λ. Fabricated for the ISM 915 MHz band, its performance is validated through simulation and measurement when implanted in a three-layer biological tissue model (skin, fat, muscle). The design achieves a balanced trade-off between miniaturization and radiation characteristics, making it suitable for wireless communication in deep-tissue implantable systems.

## 2. Antenna Topology

**Figure 1:**
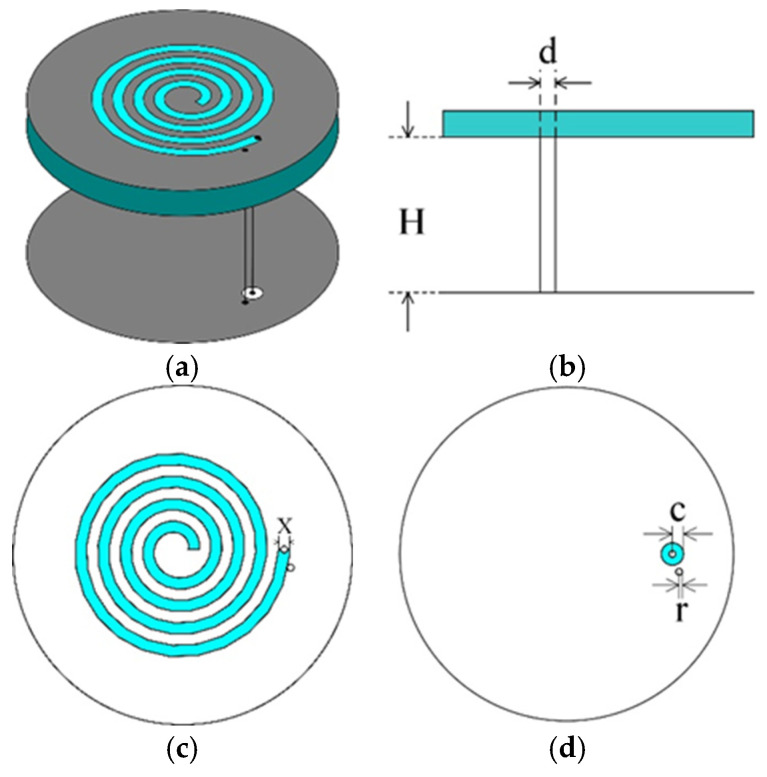
The architecture of the SSPIFA antenna. (**a**) 3D model, (**b**) front perspective, (**c**) top perspective, (**d**) bottom perspective.

**Figure 2:**
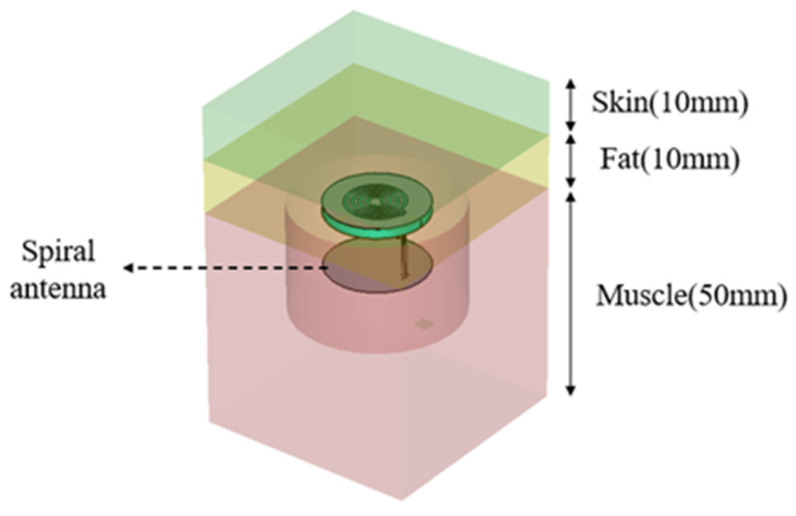
The body tissue model and the location of the SSPIFA.

The SSPIFA consists of four primary components, as illustrated in Figure 1 and Figure 2. The top layer features a circular metallic patch with a four-turn spiral slot etched concentrically, serving as the radiating element. The bottom layer is a ground plane with an identical diameter to the top patch, providing a reference for electromagnetic radiation. A metallic strip stub vertically connects the top patch to the ground plane at their centers, functioning as a reactive element to adjust impedance and enable size reduction. A coaxial feed line penetrates the ground plane through a metallic via hole, connecting to the distal end of the spiral slot to excite differential current modes along the slot perimeter. This configuration ensures compact integration while maintaining efficient electromagnetic coupling between the radiating slot and the ground plane [24,25].

The proposed SSPIFA features an ultra-compact form factor, ideally suited for scenarios demanding superior penetration capability, biocompatible safety, and conformal integration with complex multi-layered biological tissues (e.g., skin, fat, muscle laminates). A prime application is telemedicine and remote healthcare systems, where antennas are often integrated into capsule endoscopes or implantable microsensors for minimally invasive procedures. In such cases, the antenna must adhere to strict biocompatibility standards while enabling reliable wireless communication through layered tissues—requirements inherently met by the SSPIFA’s compact design and low-profile geometry [26,27,28,29,30].

A typical body tissue model, as depicted in Figure 2, is simplified as a three-layer stratified structure consisting of skin, fat, and muscle. For electromagnetic performance validation, the simulation and measurement model is defined by the following parameters: top layer (skin): permittivity (ε_r_) = 42.5, thickness = 10 mm; middle layer (fat): permittivity (ε_r_) = 5.5, thickness = 10 mm; bottom layer (muscle): permittivity (ε_r_) = 55, thickness = 30 mm. This layered configuration mimics the anatomical structure of subcutaneous tissues, providing a realistic environment to evaluate the antenna’s impedance matching, radiation efficiency, and penetration capability through biological media.

During the antenna validation process, the proposed SSPIFA is fully embedded within the muscle layer, with its spiral slot radiating patch strategically positioned to overlap the fat muscle interface, as illustrated in Figure 2. This configuration aligns the radiating element with the boundary between low-loss fat (ε_r_ = 5.5) and high-loss muscle (ε_r_ = 55), leveraging the dielectric contrast to enhance near-field coupling and mitigate tissue attenuation during wireless signal transmission.

## 3. Results and Discussion

The performance of the SSPIFA is investigated under the target application scenario, with results focusing on resonant frequency and impedance matching depth (S11 magnitude). Parametric studies are conducted to analyze how these metrics vary with key geometric parameters: antenna height, feed line width, feed stub width, and slot width. This analysis delineates the individual and combined effects of structural adjustments on the antenna’s resonant characteristics, providing design guidelines for optimizing miniaturization and impedance bandwidth in implantable environments.

### 3.1. Height

**Figure 3:**
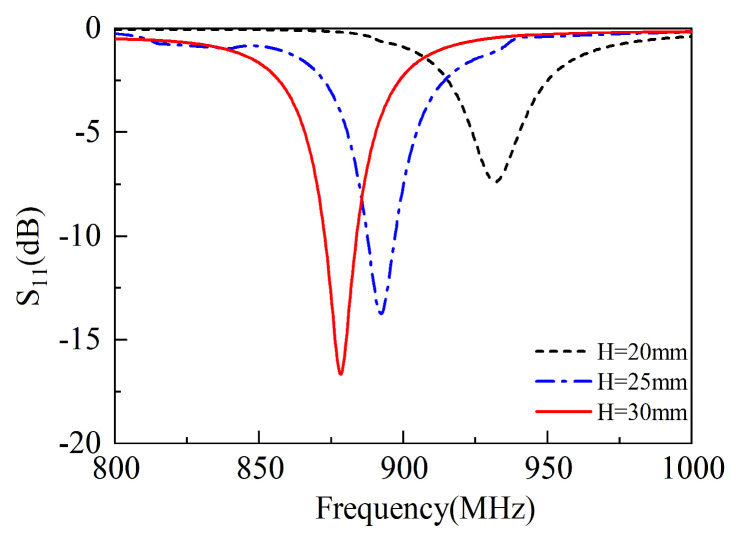
The S11 versus antenna height.

As shown in Figure 3, when H = 20 mm, the resonant frequency is approximately 880 MHz, and the resonance depth S11 reaches −17.5 dB. As H increases to 25 mm, the resonant frequency rises to about 900 MHz, and the resonance depth S11 decreases to −14 dB. When H = 30 mm, the resonant frequency becomes 930 MHz, and the resonance depth decreases to approximately −7.5 dB. This is because that the increase in H causes a change in the antenna height, resulting in an increase in frequency and a decrease in S11. Simulations show when height is increased, the frequency is reversely decreased, but the bandwidth is increased accordingly.

### 3.2. Width of the Feed Line

**Figure 4:**
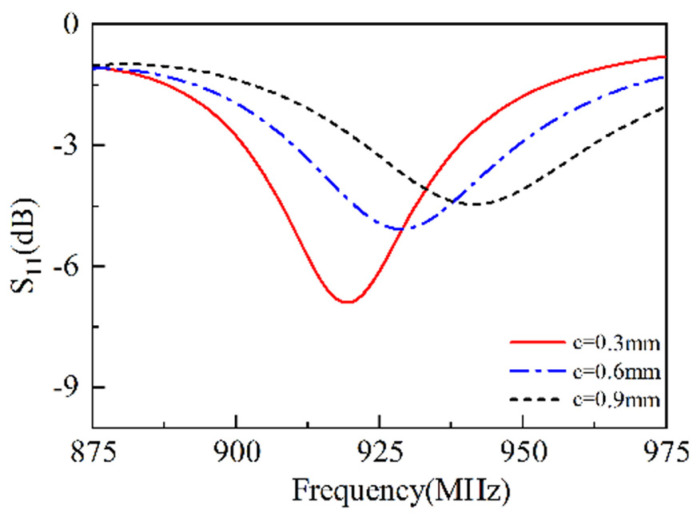
The S11 versus radius of the feed line.

As shown in Figure 4, when c = 0.3 mm, the resonant frequency is approximately 920 MHz, and the resonance depth S11 reaches −7.5 dB. As c increases to 0.6 mm, the resonant frequency rises to about 925 MHz, and the resonance depth S11 decreases to −5.5 dB. When c = 0.9 mm, the resonant frequency becomes 940 MHz, and the resonance depth decreases to approximately −4 dB. This is because that the increase in c causes a change in the radius of the feed line, resulting in an increase in frequency and a decrease in S11. The simulations show that the width of the feed line is crucial for the working frequency. When the radius is increased, the frequency is increased as well, or vice versa.

### 3.3. Width of the Short Stub

**Figure 5:**
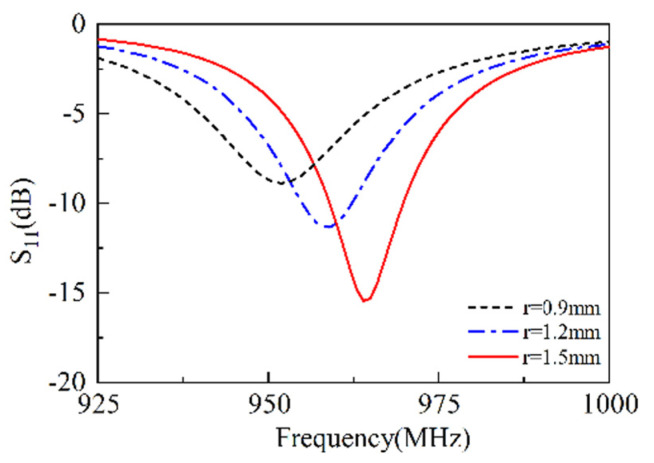
The S11 versus radius of short line.

As shown in Figure 5, when r = 0.9 mm, the resonant frequency is approximately 950 MHz, and the resonance depth S11 reaches −9 dB. As r increases to 1.2 mm, the resonant frequency rises to about 955 MHz, and the resonance depth S11 increases to −11 dB. When r = 1.5 mm, the resonant frequency becomes 965 MHz, and the resonance depth increases to approximately −17 dB. This is because that the increase in r causes a change in the radius of the short line, resulting in an increase in frequency and increase in S11. The short stub is a critical issue influencing working frequency. When the width of the short stub is decreased, the frequency is decreased as well, or vice versa.

### 3.4. Slot Width

**Figure 6:**
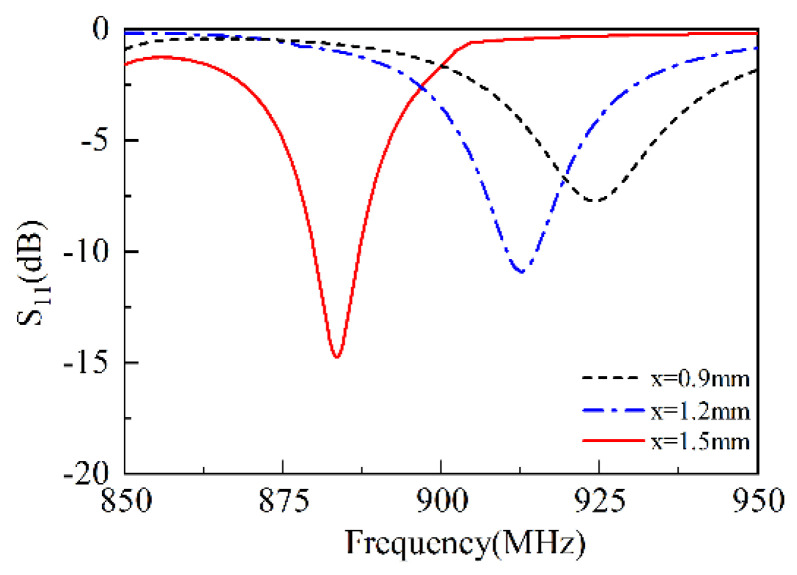
The S11 versus slot width.

As shown in Figure 6, when x = 0.9 mm, the resonant frequency is approximately 925 MHz, and the resonance depth S11 reaches −6 dB. As x increases to 1.2 mm, the resonant frequency drops to about 912 MHz, and the resonance depth S11 increases to −11 dB. When x = 1.5 mm, the resonant frequency becomes 880 MHz, and the resonance depth increases to approximately −16 dB. This is because that the increase in x causes a change in the width of the slot line, resulting in an increase in frequency and an increase in S11, demonstrating the slot width is more sensitive to the overall performance of the SSPIFA. The frequency will be increased with the increment of slot width and conversely decreased with the decrement of slot width.

## 4. Antenna Simulation and Measurement

**Figure 7:**
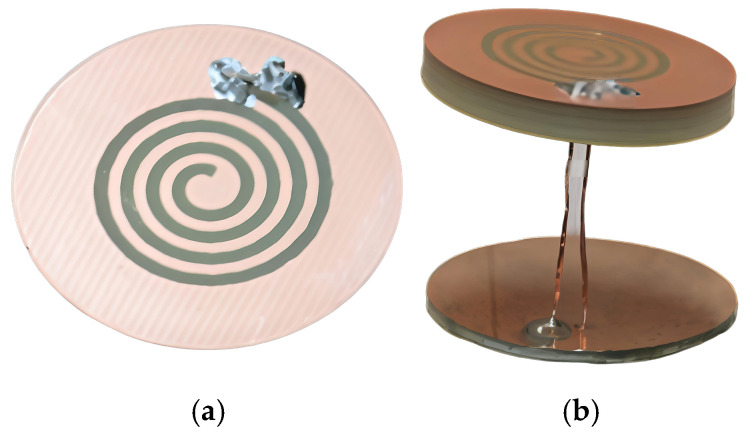
The Geometry of the SSPIFA. (**a**) the top side view (**b**) the lateral side view.

In what follows, the performance of the SSPIFA antenna is verified by simulation and measurement upon the antenna’s fabrication designed in terms of the ISM 915 MHz, a frequency said to be more appropriate for the sense of biocompatibility due to the absorption and penetrating skills. The geometric parameters of the SSPIFA antenna are a four-turns slot spiral with a slot width by 1 mm, a metallic circular top plate diameter by 30 mm on which the slot spiral is etched, and a metallic bottom plane with the same size as the top plate, both construct the outfigure of the SSPIFA with a height by 22 mm. The geometric parameters of the SSPIFA is listed in Table 1. In the antenna geometry, there is a metallic line located at one end of the slot spiral, as a feed connecting to the source through the via hole on the bottom plane, and there is a parallel metallic line, as a short line, linking the top and the bottom plane directly, as shown in Figure 7. The measurement of the antenna is implemented inside an anechoic chamber, in which the S parameter and patterns are measured.

**Figure 8:**
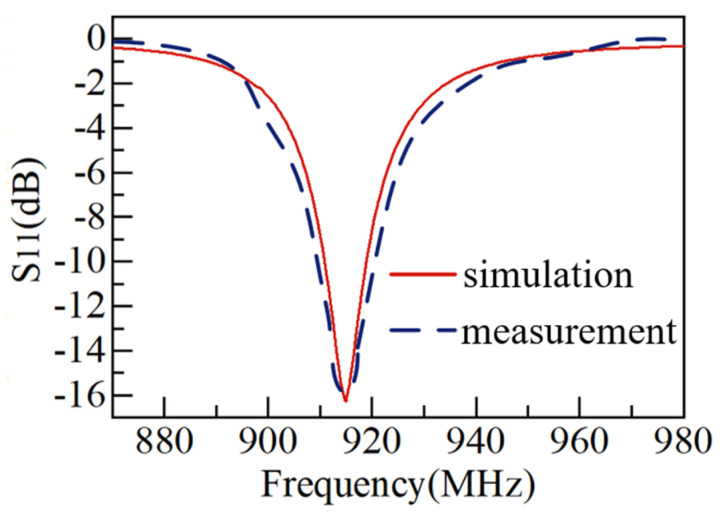
The S parameter of the SSPIFA versus Frequency.

Full-wave simulation upon the SSPIFA antenna is conducted by using the EM platform of the CST Microwave Studio, and the measurement of the antenna is implemented inside an anechoic chamber, in which the S parameter and patterns are measured. As shown in Figure 8, the S parameter shows that the SSPIFA is able to work at the 915 MHz frequency well through the indication of less than −15 dB, agreed by both simulation and measurement. The S parameter also shows that the relative bandwidth is about 1%, indicating the SSPIFA antenna is inherently a narrow circumstance for all.

**Figure 9:**
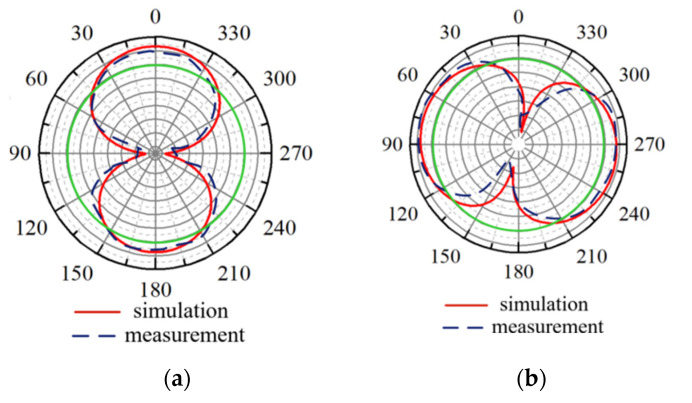
The radiation patterns at 915 MHz. (**a**) Phi = 90°; (**b**) Theta = 90°.

The radiation performance of the SSPIFA is simulated and measured as shown in Figure 8. Figure 9a is the vertical pattern (Phi = 90°), showing the gain of the antenna is 2.94 dBi; Figure 9b is the horizontal pattern (Theta = 90°), in contrast, on the horizontal plane, the pattern is more omni-direction approaching. Since the model of body tissue exist difference, which is the main reason leading to the deviation between the simulation and measurement.

**Figure 10:**
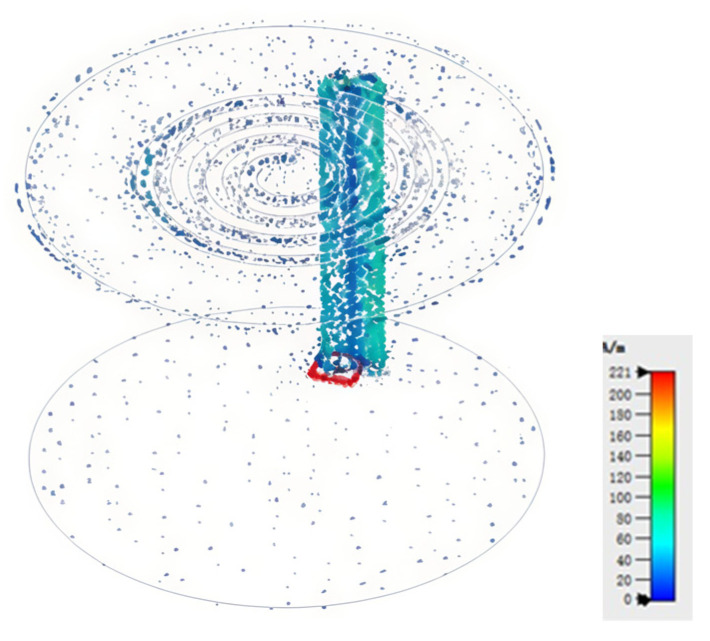
The plot of the surface current.

The surface current distribution at a frequency of 915 MHz is presented in Figure 10. The maximum surface current, reaching 221 A/m, occurs at the top of the feed line. In comparison to the ground plane, the current distributed on the top plane is significantly larger and exhibits a regular spiral type pattern, which is in accordance with the geometry of the slot spiral line.

Table 2 compares the properties and performance of the proposed SSPIFA with other reported implantable metallic spiral antennas in literature. It is evident that the proposed antenna, since using slot spiral replacing metallic spiral features with larger dimensions. The high antenna gain of the proposed antenna is achieved because it is located at the fat layer, which has low permittivity (ε_r_ = 5.5), but the compared antennas are all located at the muscle layer with high permittivity (ε_r_ = 55).

## 5. Conclusions

This paper presents the design and development of an electrically small Spiral Slot Planar Inverted-F Antenna (SSPIFA), tailored for miniaturized, biocompatible wireless solutions in telemedicine and healthcare applications. Optimized for the 915 MHz ISM band, the proposed SSPIFA features a four-turn spiral slot with a 1 mm slot width, realized on circular top/ground planes with a 30 mm diameter and an overall height of 22 mm, achieving an electrically compact profile of 0.09λ × 0.06λ.

Through electromagnetic simulations and tissue-model validations, the SSPIFA was demonstrated to reducing metallic content by replacing the conventional solid patch with a slot spiral, enabling a 30% size reduction compared to traditional PIFAs while maintaining biocompatible safety; exhibit stable performance when implanted in a three-layer biological tissue model (skin, fat, muscle), achieving a resonant S11 of −16 dB and a near-omnidirectional radiation pattern with 2.94 dBi gain in the target band.

These results validate that slot-spiral loading is an effective strategy to balance miniaturization, impedance matching, and biocompatibility in implantable antenna designs. The proposed SSPIFA holds significant value for both theoretical advancements in electrically small antenna research and practical engineering implementations in wireless medical devices, paving the way for next-generation compact implantable communication systems.

## Figures and Tables

**Table 1:** Geometric parameters of the SSPIFA.

Parameter	Definition	Value	Unit
H	Height of antenna	22	mm
D	Diameter of top & bottom plates	30	mm
c	Width of feed line	0.6	mm
r	Width of short stub	1.2	mm
x	Slot width	1.0	mm
n	Number of slot spiral turns	4	

**Table 2:** Comparison of implantable spiral PIFA antenna in literature with the proposed antenna.

Type	Frequency Band (MHz)	Size (mm^3^)	S Parameter (dB)	Gain (dB)	Substrate Material (ε_r_)
Metallic Spiral PIFA [14]	402~405	24 × 20 × 1.2	−18@403 MHz	-	10.2 (with superstrate)
Metallic Spiral PIFA [18]	402~405	π × 4 × 4 × 0.65	−24@403 MHz	−42.4 (in muscle)	9.4 (with superstrate)
Metallic Spiral PIFA [25]	600~800	π × 5 × 5 × 3.2	−19@710 MHz	−20 (in muscle)	4.3
Our work	910~920	π × 15 × 15 × 30	−16@915 MHz	2.94 (in fat)	4.3

## Data Availability

Data is unavailable due to privacy.
